# The HIV-1 gp120/V3 modifies the response of uninfected CD4 T cells to antigen presentation: mapping of the specific transcriptional signature

**DOI:** 10.1186/1479-5876-9-160

**Published:** 2011-09-24

**Authors:** Antigone K Morou, Filippos Porichis, Elias Krambovitis, George Sourvinos, Demetrios A Spandidos, Alexandros Zafiropoulos

**Affiliations:** 1 Department of Virology, Medical School, University of Crete, Heraklion, Crete, Greece; 2 Ragon Institute of Massachusetts, General Hospital, Massachusetts Institute of Technology and Harvard University, Charlestown, Massachusetts, USA; 3 Faculty of Veterinary Medicine, University of Thessaly, Trikalon 224, GR-43100 Karditsa, Greece; 4 Department of Histology-Embryology, Medical School, University of Crete, Heraklion, Crete, Greece, Voutes, Heraklion, 71409 Crete, Greece

## Abstract

**Background:**

The asymptomatic phase of HIV-1 infection is characterized by a progressive depletion of uninfected peripheral effector/memory CD4+ T cells that subsequently leads to immune dysfunction and AIDS symptoms. We have previously demonstrated that the presence of specific gp120/V3 peptides during antigen presentation can modify the activation of normal T-cells leading to altered immune function. The aim of the present study was to map the specific transcriptional profile invoked by an HIV-1/V3 epitope in uninfected T cells during antigen presentation.

**Methods:**

We exposed primary human peripheral blood monocytes to V3 lipopeptides using a liposome delivery system followed by a superantigen-mediated antigen presentation system. We then evaluated the changes in the T-cell transcriptional profile using oligonucleotide microarrays and performed Ingenuity Pathway Analysis (IPA) and DAVID analysis. The results were validated using realtime PCR, FACS, Western blotting and immunofluorescence.

**Results:**

Our results revealed that the most highly modulated transcripts could almost entirely be categorized as related to the cell cycle or transcriptional regulation. The most statistically significant enriched categories and networks identified by IPA were associated with cell cycle, gene expression, immune response, infection mechanisms, cellular growth, proliferation and antigen presentation. Canonical pathways involved in energy and cell cycle regulation, and in the co-activation of T cells were also enriched.

**Conclusions:**

Taken together, these results document a distinct transcriptional profile invoked by the HIV-1/V3 epitope. These data could be invaluable to determine the underlying mechanism by which HIV-1 epitopes interfere with uninfected CD4+ T-cell function causing hyper proliferation and AICD.

## Background

The asymptomatic phase of HIV-1 infection is characterized by the progressive depletion of uninfected peripheral effector/memory (CD45RO+) CD4+ T cells [1] that leads to subsequent immunodeficiency and AIDS symptoms. One of the potential implications of this dysfunction involves the mechanism of activation-induced cell death (AICD) that becomes enhanced and accelerated in uninfected CD45RO+/CD4+ T cells by the presence of the virus [2]. The interaction of the HIV viral envelope glycoprotein, gp120, with CD4 on the host cell surface induces conformational changes in the gp120 that allows the V3 domain of gp120 to interact with the host cell chemokine receptors, CCR5 or CXCR4 [3,4]. Although the functional importance of V3 in HIV infection has been well established [5], the effects of V3 on the host cell coreceptor signaling cascade have remained elusive through the past decade [6,7]. In CCR5-tropic HIV isolates (R5 strains), participation of the gp120 V3 domain (V3 loop) in the interaction with CCR5 is crucial for binding and cell entry [8-10]. R5 strains predominate in the asymptomatic phase, whereas isolates that utilize both CCR5 and CXCR4 (R5X4 strains) or only CXCR4 emerge much later in 40-50% of infected individuals and this often indicates the commencement to the clinical phase [11]. The persistence of an exclusive R5 viral population *in vivo *is not rare and is sufficient to cause immunodeficiency in the majority of HIV-1 infected individuals who progress to AIDS [12,13].

We have shown previously that antigen presentation can be deregulated by the presence of V3 epitopes on the surface of macrophages. CCR5 is one of the main mediators of V3-induced intracellular signaling during antigen presentation which leads to AICD, the V3-CCR5 interaction itself being of ionic nature [14,15]. Microarray studies utilizing the whole gp120 have shown increased expression of genes belonging to mitogen-activated protein kinase signal transduction pathways and genes regulating cell cycle in PBMCs [13,16].

In view of the potential involvement of V3 in the abnormal AICD process of uninfected CD4+ T cells, we addressed in this study the effects of V3 on the intracellular signaling of CD4+ T cells. We investigated the transcriptional differences in primary human CD4+ T cells attributed to the presence of V3 during antigen presentation signaling. We exposed macrophages to linear synthetic lipopeptides from the crown of V3 presented on liposomes and then we induced antigen presentation complex formation with CD4+ T cells via a superantigen presentation system [17]. Implementing oligonucleotide microarray mRNA analysis on CD4+ T cells, we assessed the impact of the V3 crown on the transcriptional state of the responding CD4+ T cells. Functional classification of significantly modulated genes and identification of canonical pathways and functional gene networks analysis were performed by an Ingenuity Pathways Analysis (IPA) platform and overrepresentation of functional ontologies by DAVID Bioinformatics Resources [18,19].

## Methods

### Peptides and liposomes

The HIV-1 gp120/V3 peptide RKSIRIQRGPGRAFY (LAI strain, a.a. 304-318) was synthesised using *F-moc*/tBu chemistry (15). Lipopeptides were produced by covalent binding of serine-S-[2,3-bis (palmitoyloxy) -(2RS)-propyl]-*n*-palmitoyl- (R)cysteine (Boehringer Mannheim Biochemica, Germany) to the V3 peptide, according to the manufacturer's instructions. Liposomes were constructed by the dehydration-rehydration method and were reconstituted with 100 μl distilled water. Non-entrapped material was removed by washing with PBS [15].

### Cell isolation

Buffy coats from healthy, HIV-1/Hepatitis b sero-negative blood donors were obtained from Venizelio Hospital Blood Transfusion Service, Heraklion, Crete. Informed consent was obtained from all the participating volunteers. Peripheral blood mononuclear cells (PBMC) were isolated by density gradient centrifugation using ficoll-paque (Amersham-Pharmacia, Uppsala, Sweden) according to the manufacturer's instructions and cultured in RPMI-1640 medium supplemented with antibiotics and 5% human serum. Depletion of CD8+ cells was carried out with the magnetic cell sorting (MACS) system (Miltenyi Biotech, Germany) using PE anti-human CD8 (Mouse IgG1, k, RPA-T8, BD Pharmingen) and anti-PE microbeads (Mouse IgG1, Miltenyi Biotech, Germany), according to the manufacturer's instructions. After the completion of the cell culture experiment, the cells harvested for the microarray analysis consisted of 86% CD3+CD4+ and 11% CD3+CD8+ double positives. This cell population will be referred to as "CD4-enriched".

### Cell culture and oligonucleotide microarray analysis

Primary cell cultures were cultivated in triplicate for 3 days with either plain liposomes (control) or liposomes with lipoV3 peptides incorporated on the liposome surface [15]. 8 hours after the addition of 1 ng/ml of staphylococcal enterotoxin A [17] (Sigma-Aldrich Chemical Co.), total RNA extraction was performed according to the manufacturer's instructions using silica-based spin columns (Qiagen RNeasy kit, Hilden, Germany). The RNA concentration was determined by measuring the absorbance at 260 nm. The purity was estimated by the 260/280 nm absorbance ratio and the integrity was assessed by agarose gel electophoresis. High quality RNA was obtained from three independently treated cell cultures from the same donor. The RNA samples were subjected to oligonucleotide microarrays by Aros Applied Biotechnology (Denmark) using the Human Genome U133 Plus 2.0 Array (Affymetrix, Santa Clara, CA, USA) according to the manufacturer's instructions. These chips contained 54,000 probe sets which could recognize transcripts from 38,500 human genes. The V3 and control RNA samples were hybridized onto three separate microarray chips each, and the values were calculated using the student's t-test to identify genes significantly modulated by V3 (p < 0.05, fold change > 2.0). Significantly modulated genes were defined as those with an absolute fold change of > 2.0 and a *P *value of < 0.05.

### Gene expression data

The primary microarray gene expression data discussed in this publication have been deposited in the ArrayExpress (European Bioinformatics Institute, EBI) database through NCBI website under accession number E-MEXP-1586, EBI http://www.ebi.ac.uk/microarray.

### Bioinformatics

We used two different bioinformatic tools to explore the functional relationships among the genes identified: the Ingenuity Pathway Analysis tool version 8.0 (IPA; Ingenuity^® ^Systems, Inc, Redwood City, CA http://www.ingenuity.com and DAVID (Database for Annotation, Visualization, and Integrated Discovery; http://david.abcc.ncifcrf.gov/) as well as extensive examination of published literature. IPA and DAVID provided complementary pathway analysis. IPA uses a proprietary knowledge base while DAVID considers ontologies from the Gene Ontology (GO) project and pathways from the Kyoto Encyclopedia of Genes and Genomes (KEGG). Functional classification of statistically significant gene expression changes was performed with IPA. This software analyzes RNA expression data in the context of known biological response and regulatory networks as well as other canonical pathways using as a reference set the Ingenuity Pathways Knowledge Base (Genes Only), filtering for molecules and relationships that are associated with the immune system. For all analyses, the right-tailed Fisher's exact test was used to calculate a P-value determining the probability that each biological function assigned to that data set was due to chance alone. Corrected p-values based on the Benjamini-Hochberg method of accounting for multiple testing were also calculated for significantly enriched Functions and Canonical pathways. Networks of highly interconnected genes were identified by statistical likelihood using the following equation:

$$Score=-\log_{10}\left(1-\overset{f-1}{\underset{i=0}{\sum }}\frac{C\left(G,\;i\right)C\left(N-G,\;s-i\right)}{C\left(N,\;s\right)}\right)$$

Where *N *is the number of genes in the network of which *G *are central node genes, for a pathway of *s *genes of which *f *are central node genes. *C(n, s) *is the binomial coefficient.

We also implemented DAVID gene functional classification to obtain biologically enriched functional gene groups with reference to our background list containing 54,000 probe sets, applying the criteria of high stringency with kappa similarity threshold set to 0.40.

### Cell cycle analysis

Progression through different cell cycle phases was monitored by flow cytometric analysis of the DNA content of cell populations stained with propidium iodide and was carried out with a fluorescence-activated cell sorter. Briefly, 48 h after the addition of SEA, cells were harvested, fixed in 70% cold ethanol for 2 hours at 4°C and stained with 20 ug/ml propidium iodide solution containing 100 ug/ml Rnase A at room temperature for 30 min. Flow cytometric analysis was performed on a FACSCalibur (Becton Dickinson) using Modfit LT analysis software (Verity Software House, Inc., Topsham, ME). A minimum of 20,000 cells was counted for each sample.

### Immunofluorescence

2*10^6 ^cells were prepared for staining using a Cytospin centrifuge (Aero spray, Wescor, USA) for 5 min at 300 rpm. Attached cells were then fixed and permeabilized, blocked in PBS containing 10% FBS, and stained with MKi67 rabbit polyclonal antibody (Abcam, ab15580) at a dilution 1:200 for 1 hour at room temperature. Cells underwent secondary staining for 1 h with Alexa488-conjugated rabbit antibody (Invitrogen, USA) at a dilution of 1:500. To-Pro 3 iodide (Invitrogen, USA) was also used for the visualization of the nuclei. The samples were observed under a confocal microscope (TCS SP2; Leica).

## Results

PBMCs were isolated from the buffy coats of volunteer healthy blood donors [20] using ficoll-paque. CD8+ T cells were negatively depleted yielding over 90% purity of CD4+ T cells. The resultant cells were aliquoted for triplicate stimulation experiments. The cells were exposed for three days to liposome constructs for V3 lipopeptides to be incorporated into macrophage cell membranes, followed by pulsing 1 ng/ml SEA [21]. Non-adherent CD4-enriched cells from each experiment were harvested 8 hours after SEA treatment and were pooled in equal proportions for RNA extraction. Transcription profiles were generated (Aros Applied Biotechnology, Denmark) with Affymetrix U133 Plus 2.0 Array chips. These chips contained 54,000 probe sets which could recognize transcripts from 38,500 human genes. The V3 and control RNA samples were hybridized onto three separate microarray chips each, and the resultant values were calculated using the student's t-test to identify genes significantly modulated by V3 (fold change > 2.0, p < 0.05). The data from the microarray study were deposited in the ArrayExpress (European Bioinformatics Institute, EBI) database through the NCBI website (accession number E-MEXP-1586, EBI). The objective of our analysis was (a) to assess genes exhibiting an extensive modulation, and (b) to identify the biological themes and functional gene clusters as a coordinated change among many gene products, while the effect of each individual gene may be subtle.

The validity of microarray results was confirmed (a) by quantitative real-time PCR (*NOC2L, IFI6, NFAT5, PI3KR1, CCNB1, TP53, MAP3K8, H-RAS*); (b) by western blotting (NFAT5, NOC2L); (c) by immunofluorescence (NFAT5, MKi67); and (d) by FACS (NFAT5, CD38).

### Genes with highly modulated expression are associated with regulation of cell cycle and proliferation

Analysis of the microarray data revealed that 440 genes were affected; 378 genes were up-regulated, 18 of which for at least 10-fold, and 62 genes were down-modulated, as compared to the control treatment. The top ranked up- and down-regulated genes are presented in Table 1. The most striking result was the 70-fold up-regulation of NOC2L mRNA, a novel gene of the HDAC-independent inhibitor for histone acetyltransferase (INHAT) [22]. A 26-fold up-regulation of the mRNA level of SEPT9 and SPIN encoding for septin 9 and spindlin 1 were also observed. NFAT5, a transcription factor that facilitates cell proliferation under hypertonic conditions was increased 18-fold due to V3 treatment. Finally, a 30-fold up-regulation of IFI6, an ISG with largely unknown function, was observed, though this result did not reach the statistical significance cut-off (p = 0.065). Regarding down-regulated genes, the gene ATP-binding cassette transporter G1 (ABCG1) that regulates cholesterol homeostasis in peripheral CD4 T cells showed the most significant down-regulation (-34.51 and -10.73 -fold). Other interesting genes that were down-regulated included PRKAR1B (-5.61-fold), which encodes the regulatory subunit of cyclic AMP-dependent protein kinase A (PKA), PPP2R1 (-2.89-fold), a constant regulatory subunit of protein phosphatase 2 and ROCK1 (-2.56-fold), a cAMP-dependent protein kinase/protein kinase G/protein kinase involved in the regulation of Fas-dependent induction [23]. As a general observation, the most significantly up- and down-regulated transcripts could almost entirely be categorized as related to the cell cycle or transcriptional regulation.

**Table 1:** Genes exhibited the highest modulation upon V3 treatment in a CD4+ T cell enriched population

Gene Symbol	Fold increase	p-value	Gene Symbol	Fold decrease	p-value
***NOC2L***	69,98	0,040	***ABCG1***	-34,52	0,019
***IFI6***	30,44	0,065	***ABCG1***	-10,73	0,026
***SEPT9***	26,67	0,038	***PGPEP1***	-7,97	0,011
***SPIN***	26,02	0,018	***PTPLA***	-7,36	0,050
***HNRPM***	18,48	0,004	***PRKAR1B***	-5,61	0,034
***NFAT5***	17,99	0,051	***FCN1***	-4,89	0,010
***NSUN6***	17,19	0,027	***SPATA21***	-4,19	0,008
***SRGAP2***	16,22	0,006	***FER1L3***	-3,79	0,015
***LOC440092***	14,93	0,002	***MYLIP***	-3,20	0,024
***GAS7***	14,77	0,008	***S100A9***	-3,12	0,047

### Multiple biological processes are affected by V3 epitope

**Figure 1:**
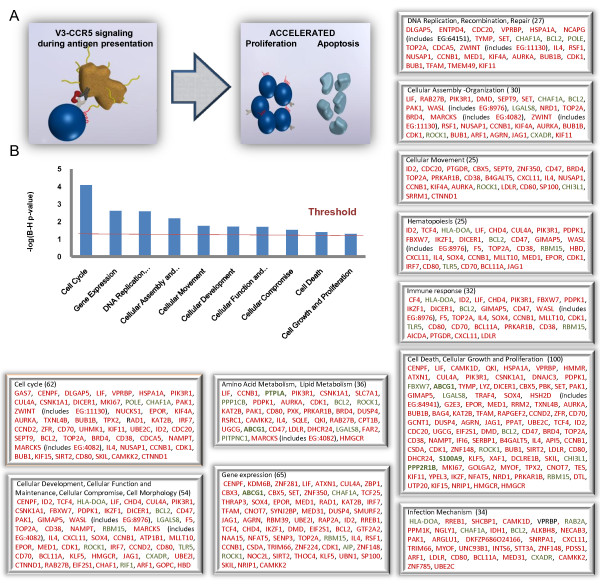
**Top molecular and cellular functions identified by IPA**. (A) IPA categorized genes exhibited significantly altered expression according to their molecular and cellular function. Green and red letters indicate decreased and increased expression, respectively. The number in the parenthesis indicates the number of altered genes classified to a certain function. (B) The diagram shows significantly overrepresented biofunctions. Corrected p-values, based on the Benjamini-Hochberg method of accounting for multiple testing, was applied by IPA to control the error rate in analysis results. A corrected p-value (FDR) of < 0.05 was considered significant.

The assignment of genes to functional categories was performed through the annotation of gene lists by IPA and DAVID that classifies genes by their ontological groups. Based on gene annotation from IPA, we classified approximately 60% of all altered genes in 29 descriptive categories of molecular and cellular functions, while 150 probe IDs (40,65%) remained uncategorized and 67 probe IDs (18,1%) did not correspond to genes (Figure 1, Additional file 1). The category of "cell cycle" achieved the highest degree of significance encompassing 52 genes (2.65E-08-2.18E-02) of which 25 genes were involved in mitosis (1.74E-04) and 5 genes in checkpoint control (*BUB1, BUB1B, CCNB1, CDC20, ZWINT*) (1.27E-01).

The second most significant category comprised of 62 genes associated with gene expression (8.34E-06-1.94E-02), of which 42 genes are implicated in DNA transcription. Other significantly enriched categories represent processes that serve the progression through cell cycle and division, such as **"**DNA replication, recombination and repair" (8.44E-06-2.18E-02) which included 27 genes associated with condensation of chromosomes, quantity of mitotic spindle and the assembly of nucleosomes, "Cellular Assembly and Organization" which included genes implicated in the missegregation of chromosomes, quantity of mitotic spindle and bundling of filaments, "Cellular Movement "(9.09E-05-2.06E-02) and particularly cytokinesis which included 25 genes and the category of "Cellular Compromise" (3.76E-04-1.94E-02) consisting of 8 genes involved in disorganization of the nucleolus and dissociation of the nuclear lamina.

**Figure 2:**
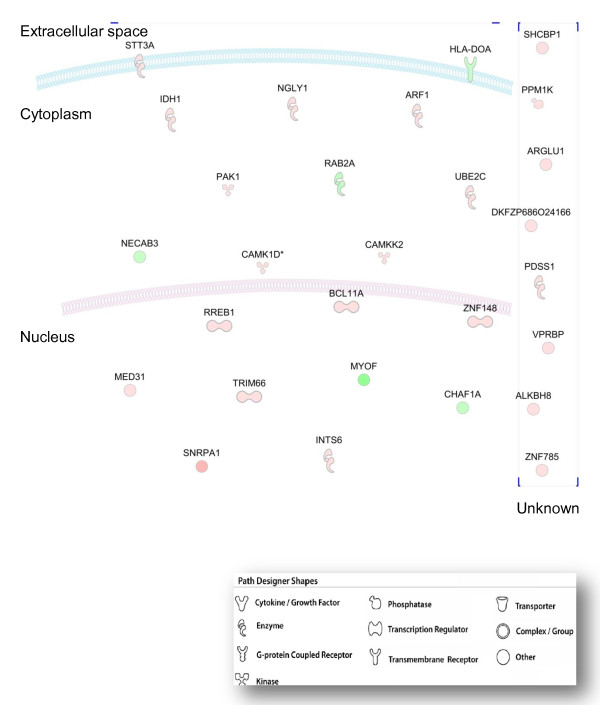
**HIV-1 infection related genes modulated by V3 epitope during the process of antigen presentation**. This diagram shows genes associated with HIV-1 infection according to IPA Knowledge Base and their cellular location. Gene products are graphically displayed as nodes. Nodes are further displayed using various shapes that represent the functional class of the gene product. The intensity of the node color indicates the degree of up- (red) or down- (green) regulation.

Of note, a number of significantly enriched categories included genes that orchestrate immune response to the V3 epitope such as " Cell-mediated immune response" (25 genes) (1.24E-04-1.94E-02), "Infection Mechanism" (34 genes) (5.82E-04-1.94E-02), "Antigen presentation" (10 genes) (1.9E-03-2.06E-02), "humoral immune response" (14 genes) (2.2E-03-2.18E-02), "immune cell trafficking" (9 genes) (2.2E-03-2.06E-02), and "inflammatory response" (10 genes) (2.2E-03-1.94E-02). Interestingly, 28 genes significantly modulated by the V3 epitope were identified by IPA to participate in HIV-1 infection (4.25E-2), of which 5 were down-regulated (Figure 2). 5 genes are involved in cell signaling (*CAMKK2, MED31, CHAF1, RREB1, PAK1*, 3.79E^-3^- 3.85E^-2^), 5 genes in post-translational modification (*PDSS1, STT3A, CAMKK2, CHAF1A, MED31*, 5.27E-3-1.05E-2), 3 genes in the cell cycle (*PAK1, CAMKK2, BRBP*) (9.2E-3- 4.9E-2) and 2 genes in lipid metabolism (*ARF1, IDH*) (1.05E-2- 3.12E-2).

The categories of "Cellular Growth and Proliferation" (1.11E-03-1.94E-02) and "Cell Death" (5.83E-04-2.11E-02) were the most abundant comprising of 66 and 81 genes, respectively. Moreover, the significantly overrepresented categories of "Cellular Development" (47 genes) (1,24E-04-2,18E-02) and "Cellular Function and Maintenance" (32 genes) (1.24E-04-1.94E-02), included mostly genes implicated in T cell development and differentiation of T lymphocytes. Other significantly enriched categories included"RNA Post-Transcriptional Modification" (11 genes) (4E-03-1.94E-02), "Protein Degradation" (6 genes) (3.86E-03-3.86E-03) and "Protein Synthesis" (8 genes) (3.86E-03-3.86E-03). Interestingly, 17 genes involved in lipid metabolism were modulated by the V3 epitope, of which 6 genes participate in the metabolism of cholesterol (*ABCG1, DHCR24, HMGCR, IL4, LDLR, SQLE*)(Additional file 2).

### Networks and canonical pathways influenced by the V3 epitope

**Figure 3:**
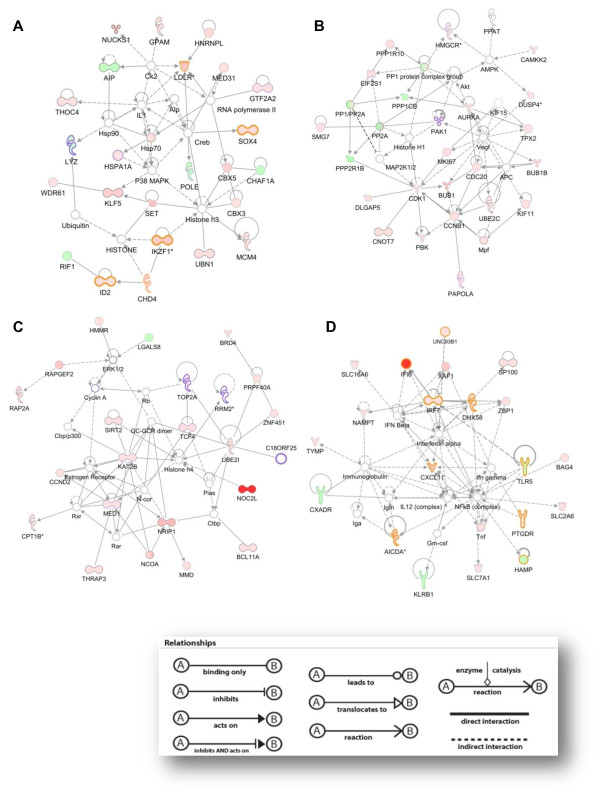
**Top ranked networks identified by IPA**. IPA networks that depict associations between genes involved in certain biological functions: (a) Gene Expression, Cellular Development, Hematological System Development and Function (score 38), (b) Cell Cycle, Cancer, Genetic Disorder (score 36), (c) Cellular Growth and Proliferation, Lipid Metabolism, Nucleic Acid Metabolism (score 35), (d) Genetic Disorder, Metabolic Disease, Antimicrobial Response (score 32). The network is graphically displayed with genes/gene products as nodes (different shapes that represent the functional class of the gene product, Figure 2) and the biological relationships between the nodes as edges (lines). The diagrams show direct (solid lines) and indirect (dashed lines) interactions between genes known to orchestrate common functions. The length of an edge reflects the evidence in the literature supporting that node-to-node relationship. Red and green shading denotes genes increased and decreased in expression, respectively, and the intensity of the colour indicates the degree of modulation. Molecules overlaid with orange and purple line are associated with immune response and HIV-1 infection, respectively. The score is derived from a *p *value and indicates the likelihood of the focus genes in a network to be found together due to random chance.

IPA generated several well-defined unique networks, consisting of 35 molecules each, out of the mapped genes that were induced in the CD4-enriched population by the V3 epitope. IPA ranks networks in order of the consistency of the microarray results with relationships confirmed by prior published results (Figure 3, Additional file 3). The first network that achieved the highest score (38), contained genes involved in gene expression, cellular development and hematological system development and function. Among these, *LDLR*, *HSPA1A*, *IKF1 *and *IDH2 *are associated with immune response and *LYZ *with HIV infection (Figure 3A). The second network contained *PP2A*, PP1 protein complex group, *CDK1*, *CCNB1 *and *VEGF *as main hubs connecting cell cycle-promoting regulators, such as *CDC20*, *BUB1*, *MKI67 *and *AURKA *(Figure 3B). The third network consisted of a number of transcription regulators and enzymes implicated in cellular growth and proliferation, and in the metabolism of lipids and nucleic acids. *NOC2L*, the gene that exhibited the most significant up-regulation was included in this network, as well as *KAT2B*, *UBE2I *and *MED31 *(Figure 3C). Interestingly, the IFNα and NFκB complex were the central hubs in the fourth network, connecting genes related to antimicrobial response, inflammation and the immune response, most notably, *IFI6*, *IRF7*, *AICDA*, *CXCL11 *and the down-regulated *TLR5 *(Figure 3D)

**Figure 4:**
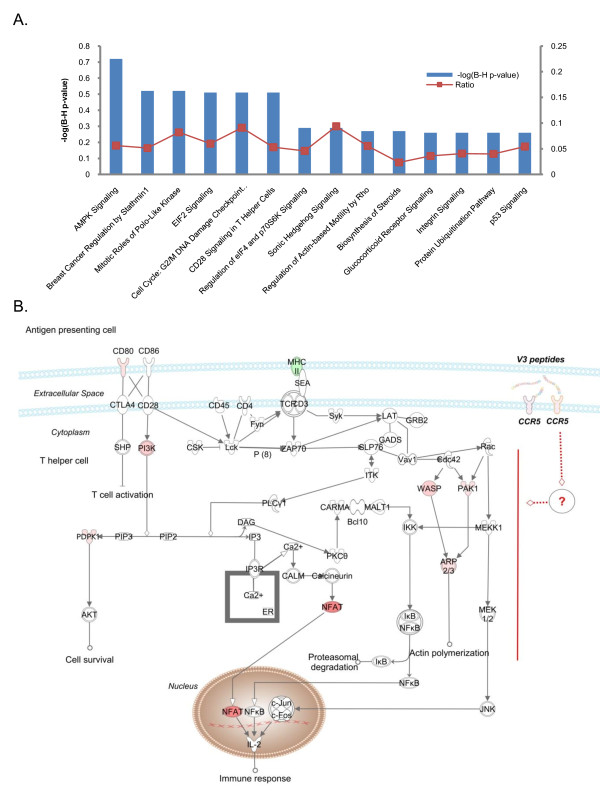
**Significantly enriched canonical pathways identified by IPA**. **(A) **The diagram shows significantly overrepresented canonical pathways. A multiple-testing corrected p-value was calculated using the Benjamini-Hochberg method to control the rate of false discoveries in statistical hypothesis testing. The ratio value represents the number of molecules in a given pathway that meet cut criteria, divided by the total number of molecules that belong to the function. **(B) **Illustration of altered genes involved in IPA canonical pathway "CD28 signaling in T helper cells". Ingenuity Pathways Analysis software was used to identify altered genes related to canonical apoptotic pathways; red and green colour indicates genes significantly increased and decreased in expression, respectively. Black indicates no significant change in gene expression.

Within IPA 23 statistically significant canonical pathways were enriched in the gene list when the corrected Fischer's exact test p-value (FDR) of < 0.05 was considered significant (Figure 4A, Additional file 4). The most significant canonical pathway identified was AMPK Signaling (FDR = 7.76E-4), the key pathway in energy regulation in which *PIK3R1*, *AK2*, *PDPK1*, *HMGCR*, *KAT2B*, *CPT1B *and *PPAT *were up-regulated, while *PRKAR1B *and *PPP2R1B *were down-regulated. Other significant overrepresented canonical pathways were involved in cell cycle progression and cell proliferation. The "Breast Cancer Regulation by Stathmin1" IPA-pathway (2.63E-3) which includes the V3-modulated genes *ROCK1, PAK1, PPP1R10, CAMK1D, PIK3R1, PRKAR1B, PPP1CB, UHMK1, PPP2R1B and CDK1*, plays a regulatory role in microtubule dynamics. In the "Mitotic Roles of Polo-Like Kinase" IPA-pathway (5.24E-3), that regulates the entry and exit in mitosis as well as cytokinesis, *CCNB1, KIF11 CDC20, CDK1 and PPP2R1B *transcripts exhibited an altered expression and in "Cell Cycle: G2/M DNA Damage Checkpoint Regulation" IPA-pathway (5.49E-3), *CCNB1*, *TOP2A*, *CDK1 *and *KAT2B *were up-regulated. The "EIF2" IPA-pathway (6.76E-3), in which the differentially expressed transcripts *EIF3B, PIK3R1, EIF4A1, PDPK1, PPP1CB and EIF2S1 *participate, regulates protein synthesis in response to various types of environmental stress and plays a crucial role in the control of the cell cycle mediating both cell proliferation and p53-dependent/independent apoptosis. Interestingly, the "CD28 Signaling in T Helper Cells" IPA-pathway, the most prominent costimulatory pathway of TCR signaling, was also significantly enriched (7.41E-3), and key molecules that participate in the pathway showed a significant transcriptional up-regulation of over 4-fold (Figure 4B). Other significantly enriched canonical pathways identified by IPA included the "Biosynthesis of Steroids" (2.39E-2), "Integrin Signaling" (2.88E-2), "p53 Signaling" (2.95E-2), "Role of Pattern Recognition Receptors in Recognition of Bacteria and Viruses" (4.89E-2) and the "ERK/MAPK Signaling" (4.89E-2) pathways.

We extended the analysis applying the DAVID Gene Functional Classification Tool, a module-centric method that takes into account the redundant and network nature of biological annotation contents in order to concentrate on the larger biological picture expressed in biological modules rather than on individual genes. Under high stringency, DAVID clustered these genes into 10 functional groups depicted in Table 2. Prominent within the list of gene clusters was the cluster of 5 molecules of the nuclear lumen and specifically of the nucleolus. The second cluster contained 7 genes implicated in nuclear division and in particular sister chromatide segregation. mRNA processing was the common function of the 12 genes in the third cluster. 5 genes involved in nuclear division were also annotated as ATP-binding phosphoproteins, and included PBK, KIF11 and AURKA, constituting the fourth cluster. The fifth cluster contained 8 genes with helicase activity. Intriguingly, 16 ATP-binding serine/threonine kinases that regulate the cell-cycle process constituted the sixth cluster. Other clusters included classified genes related to negative regulation, zinc finger, cytoskeleton and WD repeat.

**Table 2:** Ontological clustering of genes exhibiting altered expression according to DAVID

Cluster	Enrichment score	No of genes	Annotated genes	Gene ontology
**1**	7,52	5	*KRR1, NOC2L, INTS6,UTP20,YPEL3*	nuclear lumen, nucleolus
**2**	5,02	7	*DLG7, NUSAP1, ZWINT, TPX2, HCAP-G, CEP164, CDCA5*	nuclear division, M phase of mitotic cell cycle sister chromatid segregation nucleus
**3**	4,99	12	*CUGBP2, SRP46, RNPC2, HNRPL, THOC4, SNRPA1, PRPF40A, HNRPM, RSRC1, SRRM1, TRA2A, U2AF1*	mRNA processing, mRNA metabolic process
**4**	4,37	5	*PBK, KIF11, AURKA, KIF15, TPX2*	nuclear division, M phase of mitotic cell cycle, ATP binding, phosphoprotein
**5**	3,68	8	*CHD9, ASCC3L1, DDX51, PXK, EIF4A1, MCM4, LGP2, DDX24*	helicase activity, nucleotide phosphate-binding region:ATP
**6**	3,37	16	*CDC2, UHMK1, PBK, CSNK1A1, RIPK5, BUB1B, AURKA, CAMKK2, BUB1, PMSF1, MAP3K1, PSMD7, CCNB1, UBE2C, CAMK1D, CDC20*	protein serine/threonine kinase activity cell cycle process, atp-binding
**7**	2,89	5	*ZNF148, TCF25, TCF4, CSDA, ZNF281*	negative regulation of transcription from RNA polymerase II promoter, negative regulation of RNA metabolic process negative regulation of cellular biosynthetic process, transcription factor activity nucleus, phosphoprotein
**8**	2,41	9	*C13orf10, ZMYM4, ZC3H12C, ZCCHC2, SMYD4, RFWD3, BIRC4BP, ZC3H11A, KIAA0182*	zinc-finger transition metal ion binding
**9**	1,94	7	*CHD9, ASCC3L1, DDX51, PXK, EIF4A1, MCM4, LGP2, DDX24*	cytoskeleton ATP binding intracellular non-membrane-bounded organelle
**10**	1,92	5	*WDR61, SLC25A32, TP53, FBXW7, DTL*	wd repeat

Group enrichment score ranks the biological significance of gene groups based on overall EASE scores of all enriched annotation terms.

### The cell cycle state is influenced by the V3 epitope

**Figure 5:**
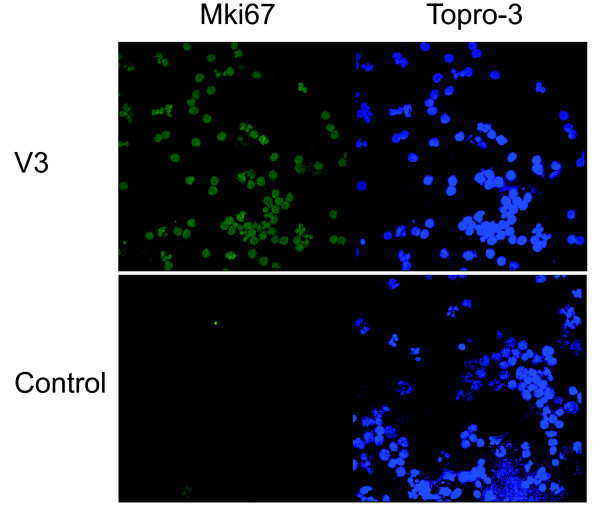
**Immunofluorescent staining of the MKi67 antigen**. Cells were exposed to V3-coated or control liposomes before activation with SEA for 24 hr (green: Alexa-488 anti-MKi67 rabbit conjugate, blue: To-Pro 3 iodide nuclear staining).

The "Cell cycle" was the most highly enriched category according our bioinformatic analysis. We validated these results with 3 independent approaches:

(a) we characterised the cell cycle state of cell populations by FACS using 3 independent donors. We observed a V3-dependent increase of cells in the S and G2/M phases in all three donors. In donor 1, we obtained a 4.1-fold increase in the percentage of cells entering the S phase and 2.01-fold increase entering the G2/M phase. The corresponding fold increases in donors 2 and 3 were 1.6 and 1.2 for the S phase, and 2.46 and 3.26 for the G2/M phase, respectively;

(b) we analysed the mRNA kinetics of *CCNB1 *by quantitative real-time PCR in 3 new donors. We observed a significant V3-dependent fold-increase which is indicative of the anticipated changes in the progression of cell cycle (additional file 5_*CCNB1*mRNA_kinetics) (c) finally, we investigated the number of MKi67 positive cells by immunofluorescence microscopy which is indicative of their proliferation state. We observed a distinct increase that further supports the impact of V3 on the cell cycle state (Figure 5).

## Discussion

The mechanism by which the uninfected CD4+ T cell population is inexorably depleted during the asymptomatic phase of HIV-1 infection remains elusive. From *in vitro *functional studies using synthetic peptides, we have concluded that the crown of the gp120 V3 loop participates in the process of antigen presentation between an HIV-infected macrophage and a responding CD4+ T cell, driving the T cell to an accelerated and enhanced AICD [17]. In the present study, we monitored changes in the transcriptional profile generated by specific signaling that is associated with the V3 epitope, in a primary CD4+ enriched population using oligonucleotide microarrays. Our results provided evidence that the V3 epitope on the surface of macrophages exerts differential and profound effects on cell cycle, gene expression, energy and metabolic demands, orchestrating the proliferation and immune response of the responding CD4+ T cells during antigen presentation.

Microarray technology and the synchronous development of bioinformatics tools that enable a more integrative and function-orientated analysis have shed some light on the immunological imprint of HIV-1 and HIV-1 accessory proteins on a variety of immune cell types [24]. Most of the studies on the putative HIV-1-induced modulation of host gene expression have been mostly performed in established human cell lines that harbor defects in crucial cellular functions, such as cell cycle, proliferation and apoptosis. This is the first study, to our knowledge, that explored the impact of a defined 15-amino acid V3 epitope of the gp120 on the transcriptional processes of physiological primary human CD4+ enriched cells. A negative selection procedure was preferred to avoid any putative antibody-mediated signaling events. Moreover, the Affymetrix chip used included genes from the whole genome and not only genes related to immune response, thus providing an integral icon of V3 impact on responding T cells. The validity of microarray results was confirmed on a set of genes from three independent donors by real-time reverse transcription polymerase chain reaction quantitative real-time PCR (qRT-PCR).

To decipher the cellular processes most affected by the V3 epitope, we classified genes with modulated transcriptional expression into (a) functional categories through IPA, and (b) into enriched gene modules descriptive of biological processes, molecular functions and cellular compartments by DAVID. Moreover, the data set was mined for networks of interconnected modulated genes, and for overrepresented pathways within the IPA library of canonical pathways.

We focused our analysis on the identification of biological themes and functional gene clusters, as a coordinated change among many gene products may produce potent biological effects, while the effect of each individual gene could be less obvious. Nevertheless, we did not underrate the role of each individual gene exhibiting an extensive modulation, as interpreting the pathways and functions of a set of differentially-expressed genes can only assess genes of known functions operating by known mechanisms. Often these well-annotated genes represent a minority of genes identified. As half of the genome is poorly annotated [25], the truly new knowledge arising from microarray experiments resides in the functions of the poorly annotated genes.

### Cell cycle control was the most overrepresented biological function among classifications of genes affected by V3

The nucleolus, which is mainly associated with ribosome biogenesis, was the most highly overrepresented gene module that emerged from DAVID analysis. An emerging body of evidence indicates that the nucleolus is a dynamic structure involved in the response to cellular stress [26,27], in regulation of cell cycle and cell growth [28] and can also be a target for virus infection [29]. Intriguingly, *NOC2L*, the most significantly mRNA up-regulated gene due to V3 treatment, is located in the nucleolus. NOC2L transcribes an inhibitor of histone acetyltransferase with a poorly defined role. Evidence suggests that NOC2L directly interacts with p53 tumor suppressor inhibiting p53-activated gene expression and p53- dependent apoptosis [22]. *NOC2L *also regulates transcription of *CDKN1A *[30], *PMAIP1 *[31] and of *TP53I3 *[32], affecting cell cycle progression and cell survival.

Prominent within the classifications were groups of genes associated with the cell cycle. Cell cycle was not only the most significantly enriched functional category, but also was the theme of the second highest network. Gene annotation clustering by DAVID highlighted the M phase of the mitotic cell cycle and specifically sister chromatide segregation and ATP binding phosphoproteins as to play crucial role on cell cycle perturbation by the V3 epitope. Canonical pathway analysis highlighted the intracellular cascades that impair cell cycle progression focusing on the regulatory role of stathmin1 (*STMN1*) in microtubule dynamics, on the mitotic role of Polo-like kinases, and on G2/M DNA damage checkpoint regulation, the second checkpoint enforced during the cell cycle. *STMN1 *is required for orderly progression through mitosis and is overexpressed across a broad range of human malignancies. Polo-like kinases are required at several key points through mitosis, starting from control of the G2/M transition through phosphorylation of CDC25C and mitotic cyclins and in the DNA damage checkpoint adaptation. Noteworthy, aberrant activation patterns of the Polo-like kinase pathway were observed in the CD4+ T cells from SIV-infected Rhesus Macaques (RM) following T-cell receptor stimulation [33]. These results imply that the V3 epitope infers cell cycle progression propelling cell division in responding CD4 T cells, a process that requires profound changes in the microtubule network that culminates in the formation of the spindle apparatus and the vigilance of DNA integrity/content by regulators of G2/M DNA damage checkpoint. The transcription of genes encoding cyclins, cyclin-dependent kinases and cell cycle regulators (*cyclin I, cyclin D2, MKI67, CDC2, CCNB1*) and the E2F cycle-specific transcription factors (*EIF2S1, EIF3S9, EIF4A1*) was enhanced presumably in order to control tightly the above-mentioned processes. This is consistent with earlier studies demonstrating that HIV-1 envelope glycoproteins expressed on T cells provoke aberrancies in cell cycle regulatory proteins, specifically accumulation of CCNB1 and hyperphosphorylation of p34cdc2 (CDK1) kinase elicited by cell-cell contact with T cells expressing CD4 coreceptors [34].

In chronically infected HIV-patients with active viral replication, cell cycle dysregulation (CCD) is associated with increased T-cell susceptibility to apoptosis and involves increased activation of the G2/M phase-associated CCNB/p34-cdc2 complex and abnormal nucleolar structure with dysregulation of nucleolin turnover [35,36]. Unscheduled p34-cdc2 activation may induce cell death by a mechanism called mitotic catastrophe [37] and CCD is consistently associated with increased levels of activation-induced apoptosis [38]. Moreover, a significantly larger fraction of CD4+ CD25+ T cells from untreated HIV+ individuals expressed cyclin A and/or cyclin B1 than was the case with those from HIV- individuals [39].

In the most significantly enriched functional categories and networks in our study, were genes associated with gene expression, DNA replication recombination and repair, and cellular development. They indicate that the V3 epitope-CCR5 interaction alters the gene activity facilitating T cell differentiation towards an immune response state. Summarizing, our data suggest that V3 signaling incites CCD in responding CD4+ T cells. This is consistent with our previous findings that the V3 epitope condemns unifected CD4+ T cells to AICD, as CCD has been associated with increased levels of AICD, and there have been observations that this V3 epitope increases the intracellular Ca^++ ^levels in responding CD4+ T cells during antigen presentation [17].

### Metabolic pathways affected by V3

The enhanced and accelerated proliferation induced by V3 presumably demands of the T cell increased energy to cope with the required amino acid and lipid metabolism. The AMP-activated protein kinase (AMPK), the most significant enriched pathway in our analysis, is a key regulator of cellular energy homeostasis and was recently shown to be activated by TCR-dependent Ca^++ ^signals [40]. Of special interest was also the up-regulation of genes involved in the synthesis of cholesterol, the basic component of lipid rafts. These highly ordered plasma membrane microdomains are reported to play a key organizing role in the formation of immunological synapses, in TCR activation and HIV infection [41]. The ATP-binding cassette transporter G1 (ABCG), a lipid transporter that promotes cholesterol efflux and regulates its subcellular distribution, was the gene that exhibited the most significant down-regulation in response to V3. Armstrong et al., in a recent study, showed that the absence of ABCG1 in CD4^+ ^T cells resulted in hyperproliferation *in vitro*, when cells are stimulated via the TCR [42].

### Genes associated with immune responses and antigen presentation were significantly affected by the V3 epitope

The presence of V3 induced increased transcription of genes that orchestrate immune response in responding CD4+ T cells, with type I interferons and NFkB being the key genes according to the IPA network. IFN-α is produced by plasmacytoid dendritic cells (pDCs) and elicits an antiviral program protecting CD4+ T cell population against HIV-1. IFN-α inhibits HIV-1 replication, formation of infectious viral particles and induces apoptosis of infected CD4^+ ^T cells. Thus, it may also account for the depletory effect of HIV on uninfected CD4+ T cells. It has been shown that when pDC and CD4+ T cells bind non-infectious HIV-1, uninfected pDC will produce IFN-α and CD4+ T cells will express TRAIL and DR5, resulting in the preferential apoptosis of uninfected T helper cells [43]. The protective/immunopathogenic balance is statistically tipped in favor of immunopathogenesis, due to the fact that non-infectious HIV-1 particles have been estimated to outnumber infectious particles.

It is worth noting that a panel of interferon-stimulated genes (ISGs) were up-regulated by the V3 epitope, namely *IFI6*, *IRF7*, *SP100 *and *IFRD1*. The integral role of these ISGs in HIV pathogenesis is underpinned by studies that corroborate the observed transcriptional up-regulation in activated CD4+ T cells of untreated HIV-positive individuals (IFI6, IRF7) [39], in an HIV-infected CD4+ T cell population derived from human tonsils (IFI6, IRF7, SP100) [44] and in lymphatic tissue of HIV-1 positive individuals throughout all stages of infection (IFI6) [45]. In addition, IFNα levels and ISG expression are increased in progressor as compared with non-progressor HIV-infected individuals [46,47]. The highly up-regulated transcriptional expression of ISGs in both AGMs, SMs and RMs during acute SIV infection persists only in pathogenic RMs during the chronic phase of the infection while it is down-regulated to baseline in AGMs and SMs [48,49]

Although NFκB did not display any significant transcriptional change, IPA network analysis attached importance to the role of NF-κΒ in regulating immune response. Transcriptional regulation by NF-κB is crucial in determining T cell fate and it has been implicated in the onset of AICD [50]. Interestingly, it has been shown that NF-κB activity is considerably enhanced after binding of NF-κB -NFAT5 complexes to κB elements of NF-κB -responsive genes [51].

In our proposed model [52], the V3 loop subverts the process of antigen presentation in order to facilitate HIV-1 entry in CD4+ T cells. "CD28 signaling in T cells", the major driver of positive immune response that elicits sustained IL-2 secretion, T cell division, clonal expansion and differentiation was conspicuous among the enriched canonical pathways. The pleiotropic impact of V3 on the CD28 signaling pathway involved the remarkable up-regulation of the transcription factor, NFAT5, the amplification of key genes acting in the PI3K/*AKT *cascade, as well as the up-regulation of three molecules that participate in CD3/CD28-triggered actin polymerization. NFAT5 is a member of the Rel family of transcription factors, which comprises of the NF-kB and NFAT proteins, which are major regulators of immune response [53]. It has been postulated that expression of NFAT5 is correlated with the duration of antigen presentation. Prolonged TCR-dependent stimulation leads to sustained activation of calcineurin and subsequent expression of NFAT5. It has also been suggested that NFAT5 participates in specific aspects of host defense by up-regulating TNF family genes that are culpable of AICD and other target genes in T cells [54]. Such mechanisms, along with the observed increased expression of NFAT5 by the V3, support our hypothesis that the interaction of V3 with the CCR5 receptor, during antigen presentation, results in persistent activation of the responding CD4 T cells leading subsequently to AICD [17].

V3 treatment also triggered the significant transcriptional up-regulation of two key genes in the *PI3K/AKT *cascade, *PI3KR1 *and *PDPK1*, that have been shown to play a role in IL-2 induced T cell proliferation. A variety of HIV proteins (gp120, gp160, Tat, Nef) impinge on pleiotropic PI3 pathway in order to facilitate HIV infection. The observed up-regulation of mRNA levels of *PI3KR1 *is reconciled with reports that associate gp120 of HIV-1 with elevated PI3-kinase activity and calcium mobilization through the chemokine receptors, CCR5 and CXCR4, in primary CD4 lymphocytes and PI-3K- mediated TNF-α production in macrophages. These studies showed that inhibition of the PI3-kinase signaling pathway suppresses virus infection post-viral entry and post-reverse transcription but prior to HIV gene expression [55,56].

The V3 epitope appeared to interfere with the CD28 signaling pathway by up-regulating *N-WASP*, *PAK1 *and *ARP2/3*, the central genes of the host cell actin remodeling machinery. Profound actin rearrangements at the immunological synapse triggered by TCR/CR28 stimulation lead to segregation of surface receptors and receptor-proximal signaling machineries to modulate, amplify and stabilize output signals to ensure ample T cell activation. It has been also proposed that gp120 modulates the synapse triggering a rapid, actin-dependent recruitment of CD4, CXCR4, and LFA-1 on the target cell during cell-cell transmission between X4 HIV-1-infected effector T cell and CD4+/CXCR4+ target T cell [57]. It is interesting to note that that Nef impairs the ability of infected lymphocytes to form immunological synapses with antigen-presenting cells lowering TCR-mediated stimulation [58]. Nef reduces actin polymerization inhibiting N-WASP that activates Arp2/3-mediated actin nucleation in lymphocytes [59]. It is tempting to speculate that Nef manipulating the formation of immunological synapses might counteract the activity of V3. To ensure its transmission, the virus needs to establish a balance between the apoptosis-prone activation state of the recipient T cell and the replication unfavorable environment of a resting T cell [60]. Thus, V3-CCR5 signaling acts as a double-edged sword; V3 renders cells hyper-responsive to stimulation favoring HIV infection and vigorous T cell proliferation, but in the case of an abortive entry of HIV into a T cell, the cell undergoes sustained proliferation but is condemned to AICD due to the absence of Nef to balance the homeostatic mechanisms of apoptosis.

Studies on the transcriptional imprint of gp120 have been previously performed in astrocytes [61], keratinocytes [62], natural killers [63], pDCs [58] MMDCs [64] and PBMCs [16]. In these studies, gp120 generated subtle changes in the transcriptional profile of astrocytes and induced apoptotic genes of the Fas/FasL pathway in keratinocytes. In NK cells, that lack CD4 receptor, gp120 signaling through CCR5 elicited up-regulation of genes involved in apoptosis and down-regulation of genes involved in proliferation and survival. In pDCs, gp120 interfered with the transcription of genes associated with TLR9 activation and decreased the ability of pDCs to secrete antiviral and inflammatory factors, while in monocyte-macrophage dendritic cells, no significant effect of gp120 on the transcriptional state was found. The transcriptional changes by gp120 in these type of cells were less profound than the modifications in T cells, which is consistent with the fact that these type of cells are less conducive to HIV-1 infection. In an elegant study by Cicala et al [16], in which the effect of R5 and X4 gp120 on PBMCs was discriminated, it was shown that R5 gp120s exerted a greater impact on the transcriptional profile of responding cells than X4 gp120, and the cell cycle and proliferation were among the gene categories strongly modulated by V3. Interestingly, treatment of cells from CCR5Δ32 homozygous donors with gp120 derived from an R5 virus, demonstrated that the majority of responses required the engagement of the CCR5 coreceptor. Although in the present study we investigated V3-specific signals in a CD4+ enriched population, our findings are consistent with those reported by Cicala et al.

## Conclusions

V3 re-immerged as a promising target for neutralizing antibodies when the 3D structure was recently revealed. However, its precise role in HIV infection remains elusive. In this study, we showed that the crown of V3 imposes a strong transcriptional signature pattern on responding CD4 T cells during antigen presentation associated with cell cycle, gene expression, immune response, infection mechanisms, cellular growth, proliferation and antigen presentation Unveiling the key participating molecules in intracellular signaling within the responding CD4 T cells may provide the impetus to design novel therapeutic strategies such as the salvaging of uninfected T cells from HIV-induced AICD.

## Competing interests

The authors declare that they have no competing interests.

## Authors' contributions

AM: performed the in vitro experimental protocol and bioinformatic analysis of microarray data, validation of selected microarray results by Real-time PCR, immunofluorescence, western blotting, FACS in independent donors, cell cycle analysis by FACS, and prepared the manuscript; FP: applied jointly with AM the *in vitro *experimental protocol and designed the microarray experiment; EK: as Principle Investigator, supervised the whole project and gave the final approval to the manuscript. GS and DS: participated in the design of the project and critically reviewed the manuscript; AZ: assisted in the design and supervision of the experiments, and in the preparation of the manuscript;

All authors read and approved the final manuscript.

## Supplementary Material

Additional file 1 **Gene annotation of IDs mapped by IPA**. Click here for file

Additional file 2 **Overrepresented functions identified by IPA**. Click here for file

Additional file 3 **Networks identified by IPA**. Click here for file

Additional file 4 **Enriched canonical pathways identified by IPA**. Click here for file

Additional file 5 **CCNB1 mRNA_kinetics**. Click here for file
